# Effect of Pulsed Current-Assisted Tension on the Mechanical Behavior and Local Strain of Nickel-Based Superalloy Sheet

**DOI:** 10.3390/ma16041589

**Published:** 2023-02-14

**Authors:** Yuxi Chen, Jie Xu, Bin Guo, Debin Shan

**Affiliations:** 1 Key Laboratory of Micro-Systems and Micro-Microstructures Manufacturing of Ministry of Education, Harbin Institute of Technology, Harbin 150080, China; 2 School of Materials Science and Engineering, Harbin Institute of Technology, Harbin 150001, China; 3 National Key Laboratory for Precision Hot Processing of Metals, School of Materials Science and Engineering, Harbin Institute of Technology, Harbin 150001, China

**Keywords:** nickel-based superalloy, pulsed current, local strain, Joule heating

## Abstract

Electrically assisted (EA) forming is a plastic forming technique under the coupling action of multiple energy fields, such as force field, temperature field, and electric field. It is suitable for the forming of difficult-to-deform materials such as nickel-based superalloys. In this paper, uniaxial tensile tests on nickel-based superalloy sheets were carried out using the pulsed current assisted with different parameters. The experimental results show that the flow stress of the material decreased with the increase in the current density under a high-frequency pulsed current, and the Joule heating effect explains the flow stress drop. In the pulsed current application process, the different types of Portevin–Le Chatelier phenomena appeared with the increase in the current density. The decrease in elongation assisted by the pulsed current was explained by analyzing the inhomogeneity of the maximum Joule heating temperature distribution. In addition, the digital image correlation (DIC) analysis was used to analyze the local strain behavior of the pulsed current-assisted tensile process. Under the application of a high-frequency pulse current, the specimen exerted an inhomogeneous temperature increase and local hot pressing stress, which resulted in the inhomogeneous distribution of the local strain.

## 1. Introduction

In recent years, micro-electromechanical systems (MEMS) and microsystem technology (MST) have gradually developed towards miniaturization, integration, and intelligence. Thin plate micro components occupy an important position. However, these vital structural components have strict material requirements and need excellent service performance in high temperatures, high pressure, and particular corrosive media. Nickel-based superalloy still has excellent mechanical properties and fatigue, oxidation, and creep resistance under high-temperature conditions, as well as good corrosion resistance and welding performances. Nickel-based superalloys have become one of the most widely used for the hottest components, such as modern aircraft engines, gas turbine disks, and generators [1,2,3].

As a highly alloyed material, nickel-based superalloy has high deformation resistance after solid solution strengthening and precipitation strengthening. A significant work hardening phenomenon results in decreased plastic deformation ability during room temperature forming. How to obtain the excellent service performance of nickel-based superalloys while preparing thin plate microstructural parts with high precision, high quality, low cost, and mass production has become a key issue to be solved urgently. According to X. Liu et al., a high-temperature hot working assisted forming process can effectively reduce the yield strength and work hardening of nickel-based superalloys [4]. However, the traditional high-temperature hot forming process has the disadvantages of difficulty in demolding, reducing the service life of the mold, and increasing energy loss. New energy field forming technology has developed rapidly, including electrically assisted forming. Electrically assisted forming (EAM) applies electricity to the blank during the plastic forming process. EAM technology has become a research topic of great interest. The EAM of micro components has great significance in the fields of aerospace, equipment, engines, and energy. By investigating the elastoplasticity of aluminum alloy under a pulse current, Jae-Hun Roh et al. found that tensile specimens’ formability was significantly improved with pulse current assistance [5]. Moon-Jo Kim et al. reported that the flow stress of the metal was softened and the elongation was significantly improved under the EA tensile test. In the microstructural observation of the metal, the dislocation recovery was found at a specific current density, which proved that the current plays an important role in the process of induced annealing [6]. The plasticity of nanocrystalline nickel foil under pulse current treatment is significantly improved by grain boundary sliding, formation of deformation twins, and dislocation sliding. The elongation is significantly enhanced compared to nanocrystalline nickel foil without pulse current [7]. Jinlan An et al. found that the increase in atomic thermal vibration in in situ pulsed current leads to the decrease in Peierls’s stress, which is the essential factor for reducing flow stress [8]. Xin Zhang et al. used in situ transmission electron microscopy to directly observe the dislocation evolution process of superalloys under the action of electric current, proving electronic force’s existence in the EA manufacturing process [9]. Y. Z. Liu et al. demonstrated that EA could suppress the grain size effect of superalloys by conducting room temperature and EA tensile experiments on nickel-based superalloy sheets and established a multiscale constitutive model considering various strengthening mechanisms under EA [10]. The EA process has also made considerable progress. In the process of EA rolling, S. X. Xue et al. found that a higher forming height can be obtained under appropriate current parameters, and the foil thickness change is significantly reduced [11].

EA forming has been widely studied by scholars at home and abroad in metal forming performance. However, in EA difficult-to-deform materials (such as nickel-based superalloys), the mechanism of the deformation behavior under different current parameters needs further detailed investigation. In addition, the local strain distribution of EA forming in the forming process is rarely studied. Therefore, it is meaningful to investigate the effect of the current on the local strain of materials. In this paper, high-frequency pulse current-assisted tensile tests were carried out on a nickel-based superalloy GH4169 sheet after solution treatment. The effect of the high-frequency pulse current assistance on the mechanical properties of thin plates under different current densities was studied. The real-time maximum joule heating temperature changing in the thin plate deformation region with high-frequency pulse current assisted was investigated by infrared imager measurement. The digital image correlation (DIC) technique was used to observe the local strain evolution to clarify the effect of the high-frequency pulse current on the strain distribution in the tensile process of nickel-based superalloy sheets.

This paper aimed to clarify the regulation and mechanism of pulsed current on the tension of nickel-based superalloy. It provides a theoretical basis for potential applications, such as forming [12,13,14] and cutting [15,16]. It has guiding significance for green, high-precision, and controllable forming of difficult-to-deform materials.

## 2. Materials and Method

### 2.1. Experimental Equipment

**Figure 1:**
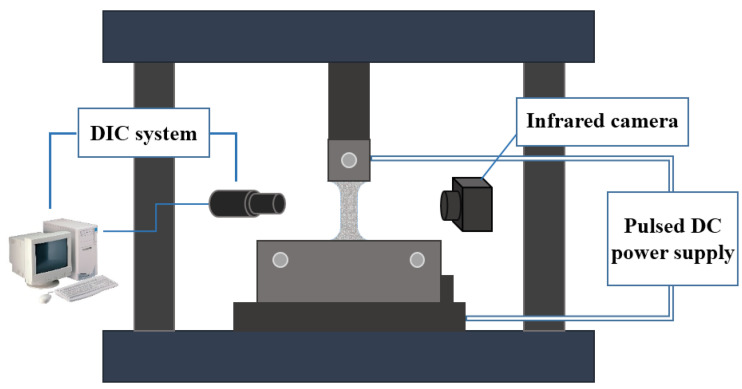
Pulsed current-assisted tensile device.

The experiment was carried out on an electronic universal testing machine (AGXplus-50 kN, Shimadzu, Japan), with a constant strain rate of 0.0075 mm/s, i.e., strain rate of 10^−3^ s^−1^. The fixture connector connected the electronic universal testing machine and the current-assisted tensile fixture. In order to limit the pulse current to the sample area, an insulation plate was used to conduct the insulation treatment between the mold and the sample on the inside of the clamping end of the mold. The two poles of the pulse power supply were connected to the copper plates on both sides of the mold clamping end sample through the copper wire. This paper used a programmable pulse power supply(MicroStarCRS-LFP20-500, Dynatronix, Amery, WI, USA) to realize the output of the direct current (DC) and pulsed current. The control panel of the pulse power supply, the output current size, pulse time and period, and duty ratio could be adjusted by programming. The pulsed current-assisted tensile device was shown in Figure 1.

In the pulsed current-assisted tensile test, it was necessary to measure the temperature at the specimen gauge. The temperature acquisition system was composed of a infrared thermal imager (FLIR T660 Imager, FLIR Systems, Wilsonville, OR, USA) and a computer. The temperature acquisition system could realize noncontact full-field real-time temperature measurement at the gauge distance of the tensile specimen.

In addition, the strain of the sample was measured by the noncontact DIC system during the pulse current-assisted tensile test. The DIC measurement system included a CCD camera (GS3-U3-15S5M-C, FLIR Systems, Wilsonville, OR, USA), lens, light source, speckle tool, image acquisition software Vic-Snap (Version 8, Correlated Solutions, Columbia, SC, USA), and image analysis software VIC-2D (Version 6.0.2, Correlated Solutions, Columbia, SC, USA). The lens was adjusted to the sample gauge position using an XYZ platform mount during the current-assisted tensile test.

### 2.2. Experimental Material

**Figure 2:**
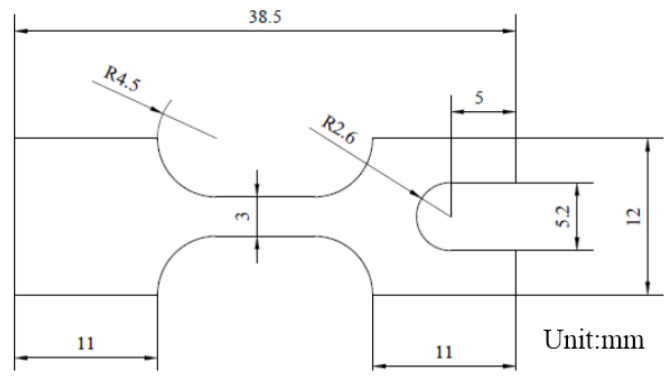
GH4169 sheet specimens and dimension.

The material used in this paper was GH4169 (52.14Ni-18.54Cr-3.1Mo-4.77Nb-1.11Ti-0.64Al-0.28Si-Fe (wt%)) nickel-based superalloy sheet rolled by Beijing Institute of Iron and Steel. The thickness of the thin plate was 200 μm. The tensile specimen was processed by electro-discharge wire cutting along the rolling direction of the sheet, as illustrated in Figure 2, where the gauge width was 3 mm, and the gauge length was 7.5 mm.

**Figure 3:**
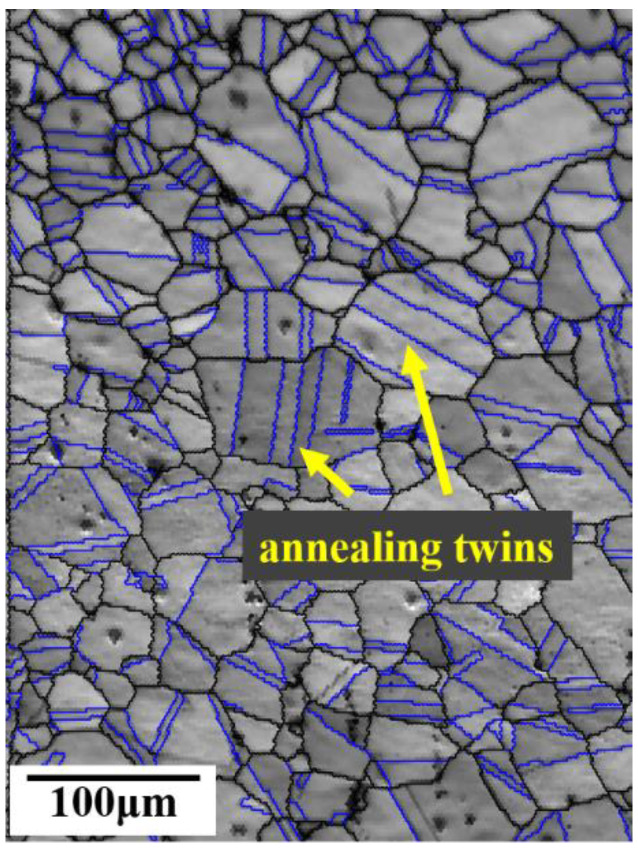
Grain map of the alloy subjected to the solution treatment.

Before the tensile test, the tensile specimen needed to be treated by solution treatment, and the solution treatment adopted T1260, a high-temperature vacuum tube heat treatment furnace. The heating rate was set to 10 °C/min, raised to 1050 °C after 1 h, and water cooling was chosen for the cooling. The grain map of the solution-treated specimens is depicted in Figure 3. After the solution treatment at 1050 °C, the average grain size was 44.22 μm. The grain size was measured using the intercept method according to ASTM E112. The grains after the solution treatment were composed of austenite equiaxed grains, and there were a certain number of annealing twins.

## 3. Results and Discussion

### 3.1. Effect of High-Frequency Pulsed Current on the Tensile Mechanical Properties of Specimens

#### 3.1.1. Effect of High-Frequency Pulsed Current on the Flow Behavior

**Figure 4:**
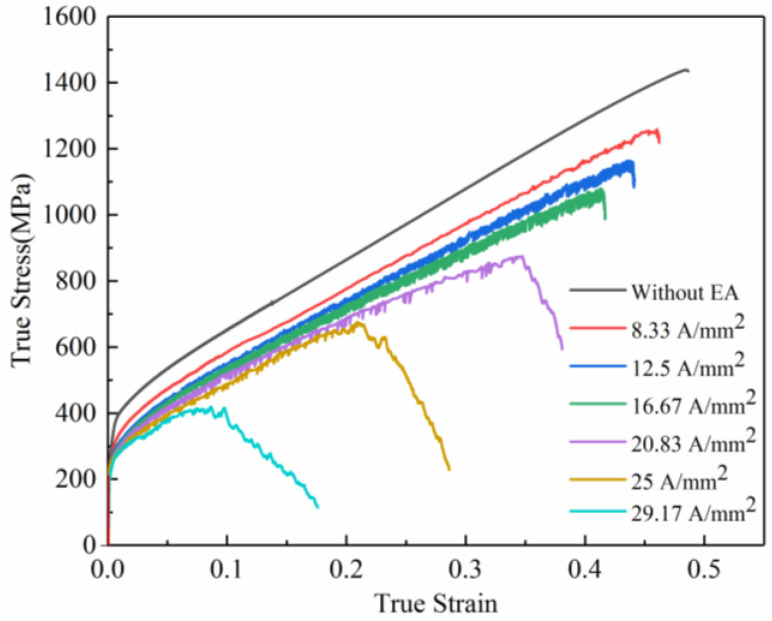
True stress–true strain curves of the specimens with different current densities.

The tensile tests assisted by high-frequency pulsed currents under different current density parameters were carried out on the GH4169 superalloy sheet. The power parameters were set as follows: pulse period 0.01 s, pulse width 0.005 s, and frequency 100 HZ. In this paper, the experiments were carried out five times for each parameter. Figure 4 shows the true stress–true strain curves of the specimens with various current density parameters. It is seen that the flow stress of tensile specimens with all current densities has different degrees of reduction after inputting the high-frequency pulsed current. In addition, with the increase in the current density, the flow stress decreased more obviously. The current-assisted tension caused stress softening of the specimens. This phenomenon was mainly caused by the Joule heating effect and the electronic force caused by the applied electric field. Due to the resistance of metals, the Joule heating effect will be produced when the current is introduced into the specimen, which will increase the specimen’s temperature and decrease the deformation resistance [17,18]. The electronic force also has a significant effect on the reduction of material flow stress. Nonthermal electron wind is a typical mechanism to explain the electron force effect [19,20]. The nonthermal electron wind reduces the flow stress of the material by the interaction between electrons and dislocations [21]. This was verified by the electrical in situ TEM [9]. In addition, the current also has an effect on the microstructure. The current can reduce dislocation pile-up and promote dislocation slip. Moreover, the dislocation slip is more significant with the increase in the current density [17]. Our group established a flow stress model including the effects of strain hardening, rate hardening, thermal softening, solute–dislocation interaction, and electron wind. The modeling results demonstrate that Joule heating is the major factor affecting the deformation behavior under microtension subjected to current [22]. When the current density was lower than 16.67 A/mm^2^, the stress–strain curve shape was similar to the curve without current-assisted tensile test. However, when the current density was higher than 20.83 A/mm^2^, the flow stress increased first with the increase in the strain and then decreased significantly until the specimen broke.

**Figure 5:**
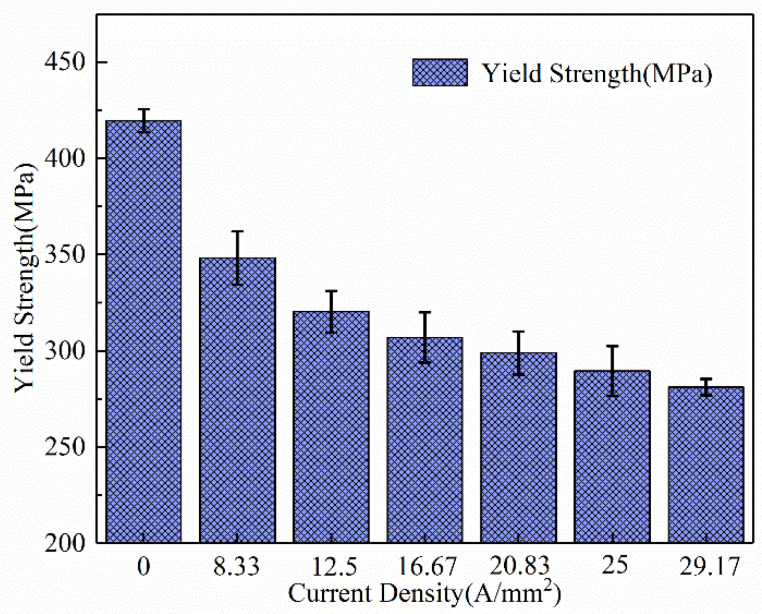
Effect of the high-frequency pulse current on yield stress.

**Figure 6:**
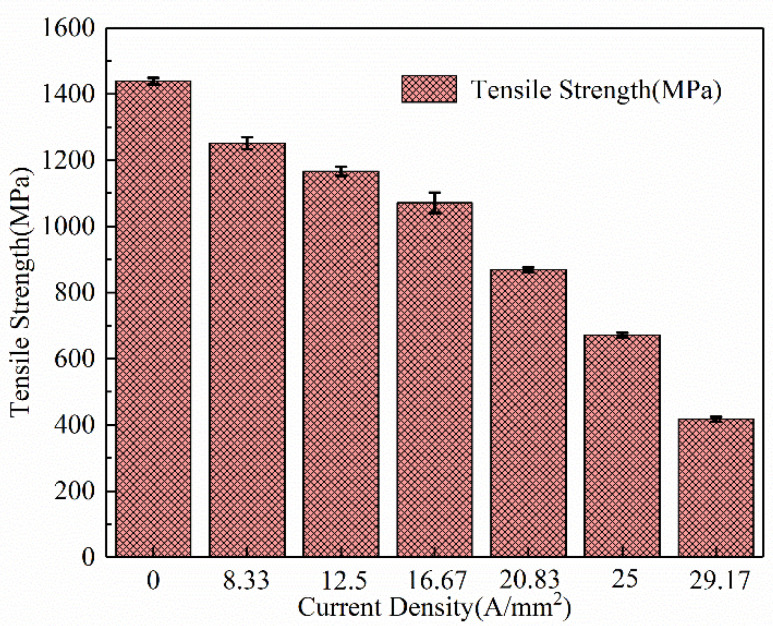
Effect of the high-frequency pulse current on tensile stress.

The yield strength and tensile strength of the specimens under high-frequency pulse current-assisted tension with different current densities are illustrated in Figure 5 and Figure 6. It can be found that the yield strength and tensile strength of the material decreased with the increase in the current density. Compared with the yield strength of the specimen without pulsed current assistance, the yield strength of the specimen with pulse current assistance was significantly reduced. With the increase in the current density, the yield strength of the specimen decreased slightly. The tensile strength of the specimen decreased more significantly with the increase in the current density. Reasonable control of the current density can considerably reduce the deformation resistance while ensuring the material properties.

**Figure 7:**
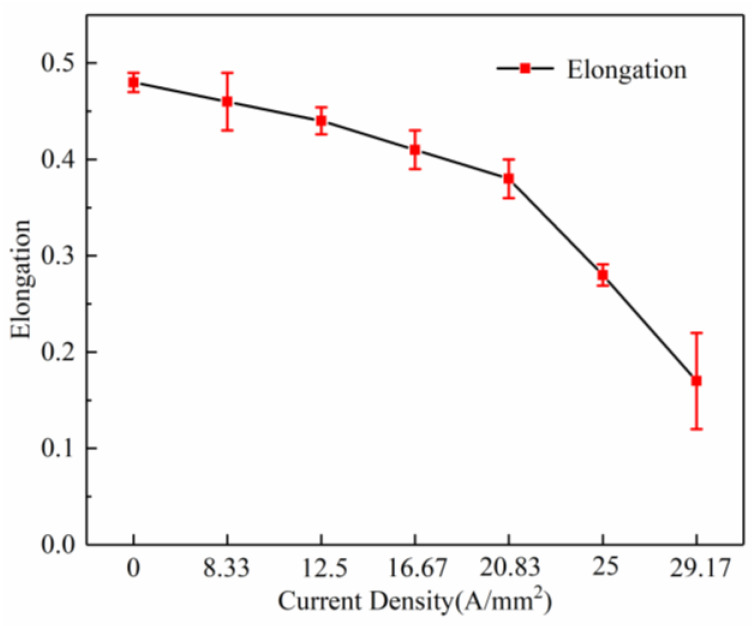
Effect of the high-frequency pulse current on elongation.

The elongation of the specimens under high-frequency pulsed current-assisted tension with different current densities is shown in Figure 7. It was found that the elongation of the specimens with different current densities decreased. It has been known that the application of pulsed current can significantly enhance the formability of metals [5,23]. It was reported that the defect configuration can be dramatically modified by the application of pulsed currents, from localized planar slip to homogeneous wavy slip. This mechanism enhances the strength and ductility by changing the dislocation pattern during deformation [24]. However, it has also been reported that the elongation of different materials decreases with the increase in the current density under pulse current [10,20]. Kim, Moon-Jo [25] found that the current accelerated the formation of microvoids. The microvoids might considerably reduce the bonding force between particles and the matrix, which induces earlier fracture in the specimen subjected to the pulsed electric current.

**Figure 8:**
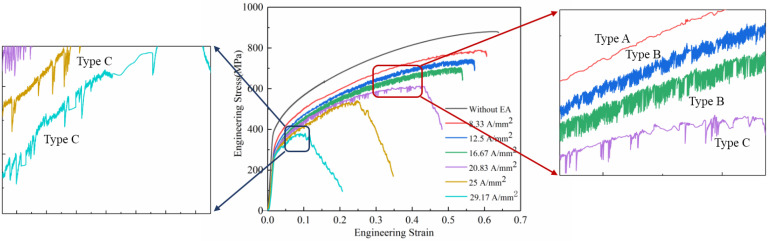
Effect of the high-frequency pulse current on the PLC morphology.

The engineering stress–strain curves of the specimens under different current densities are shown in Figure 8. During the current-assisted tension, a serrated flow stress curve different from the conventional tension appeared, which is the Portevin–Le Châtelier (PLC) phenomenon that occurred. This phenomenon was due to the diffusion of solute atom interaction with the dynamic pinning and release of moving dislocations during plastic deformation [26]. In addition, the three serrated shapes in the stress–strain curve of Figure 8 correspond to three types of PLC effects. When the current density was 8.33 A/mm^2^, the sawtooth shape of the curve was the type A PLC. According to P. Maj, the type A PLC stress fluctuations were not evident and irregular. The sudden increase in the flow stress was caused by the pinning of solute atoms to moving dislocations [27]. When the current density was 12.5 and 16.67 A/mm^2^, the sawtooth shape was the type B PLC, and the flow stress oscillated rapidly with the increase in the strain. It was reported that type B serrations were usually associated with the PLC effect based on the dynamic strain aging (i.e., diffusing solute atoms pinning dislocations in place) [28,29]. When the current density was 20.83, 25, and 29.17 A/mm^2^, the sawtooth shape was the C-type PLC, and the sawtooth had no regular oscillation below the stress–strain curve. The reason for the appearance of C-type PLC is that the solute atoms have a strong enough diffusion ability under low strain, (i.e., the dislocation is pinned at the beginning of the deformation). The dislocation is released under the applied stress with strain increases, resulting in a decrease in the stress [26].

The PLC type of the tensile specimen was obviously not the same at different current densities. The reason might be that different current densities affect the diffusion ability (vibration ability) of solute atoms which, in turn, affects the interaction between diffused solute atoms and mobile dislocations.

**Figure 9:**
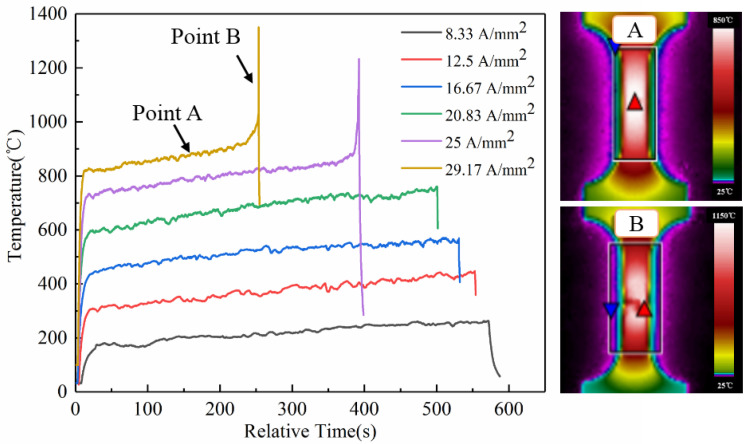
Temperature variation in the specimens with different current densities. (Point A and B are the infrared thermal imager figures of the specimens in the gently rising stage and the instantaneous rising stage under the current density of 29.17 A/mm^2^).

The infrared imager was used to measure the real-time temperature of the gauge part during the deformation of the specimen. The maximum Joule heating temperature change at the gauge of the specimens was observed, as shown in Figure 9. The temperature of the specimens increased rapidly at the moment of the high-frequency pulse current, then it increased slightly over time. The temperature fell off a cliff after the specimens broke. The temperature increase curves at different current densities were basically similar. In addition, as the current density increased, the maximum Joule heating temperature increased. At high current densities (25 and 29.17 A/mm^2^), the instantaneous temperature rise occurred before the material breaks. 

Therefore, the maximum Joule heating temperature curve points A and B at the current density of 29.17 A/mm^2^ were selected. In the maximum Joule heating distribution for points A and B, the red triangle was the maximum Joule heating position in the figure. It was found that the Joule heating temperature was the highest in the central region of the specimen gauge length and gradually decreased from the middle to the boundary. The point B was where the maximum Joule heating temperature curve broke after the instantaneous rise, and the maximum Joule heating position was still the center of the gauge length. The Joule heat temperature was the highest in the gauge center area. After the pulse current was introduced, the heat concentration was generated after necking, resulting in a more serious strain concentration, which led to a decrease in the elongation.

#### 3.1.2. Effect of High-Frequency Pulsed Current on Fracture Morphology

**Figure 10:**
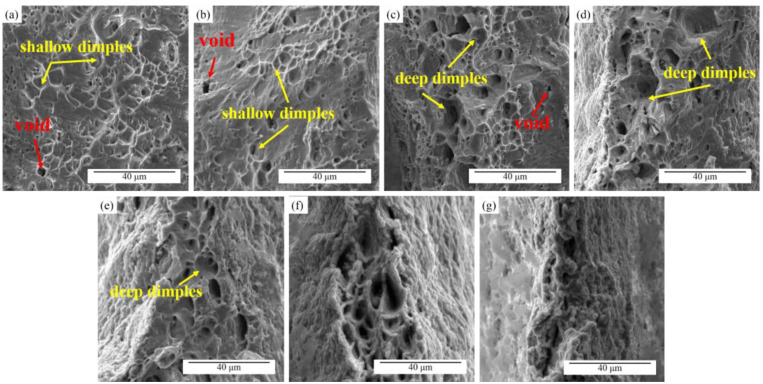
Fracture morphology: (**a**) without EA; (**b**) 8.33; (**c**) 12.5; (**d**) 16.67; (**e**) 20.83; (**f**) 25; (**g**) 29.17 A/mm^2^ tensile specimens.

The fracture of the specimens without high-frequency pulse current assistance had typical ductile fracture characteristics, as shown in Figure 10a. There were many small equiaxed dimples in the fracture morphology of the specimens. These equiaxed dimples were traces left on the fracture surface by the nucleation, growth, and aggregation of voids under external force. At the smaller current density (8.33 A/mm^2^), there was no noticeable difference in the fracture morphology of the specimens. However, when the current density increased, as shown in Figure 10c–e, a part of the larger and deeper dimples appeared in the fracture morphology in addition to the shallower equiaxed dimples, and there was a high ridge between the dimples. Due to the electric current induced void nucleation and growth around the carbides, the “bracket-shaped” voids with lager volume formed. Under the current-assisted tension, unique bracket-shaped voids formed, which might be the reason for the formation of the larger and deeper dimples [30]. When the current density was 25 and 29.17 A/mm^2^, there were different degrees of local melting on the fracture surface of the specimens. It was presumed that the instantaneous increase in the temperature at the necking of the specimens before the fracture led to the melting of the dimple boundary.

### 3.2. Effect of High-Frequency Pulsed Current on the Tensile Strain Distribution of the Specimens

#### 3.2.1. Effect of High-Frequency Pulsed Current on the Strain Surface Distribution

**Figure 11:**
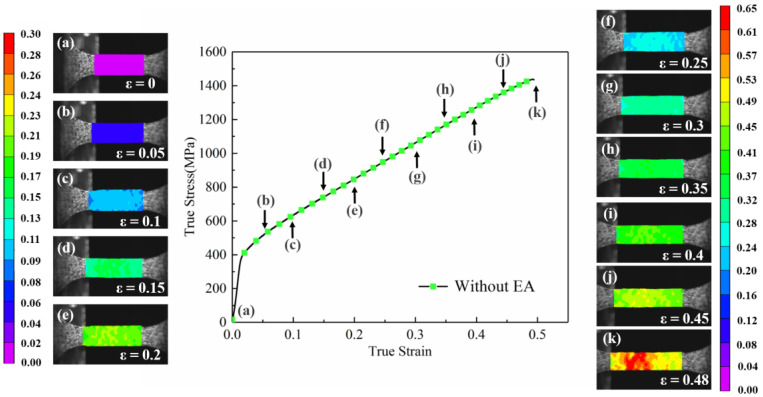
Strain surface distribution along the specimen’s tensile direction without high-frequency pulse current assisted. (**a**–**k**) are the surface distribution figures under macro strain from ε = 0 to ε = 0.48.

The DIC optical measurement method was used in this paper to investigate the evolution of the local strain distribution of superalloy sheets in the high-frequency pulse current-assisted tension. The DIC analysis area was the gauge part of the specimen. The full-field strain distribution on the surface of the tensile specimen was obtained by image analysis software VIC-2D. Figure 11 shows the strain surface distribution of the specimen along the tensile direction without the assistance of the high-frequency pulse current. Figure 11a–k are the surface distribution figures under different macro strains in the corresponding figure, from strain ε = 0 to strain ε = 0.48 before fracture. Because the specimen surface distribution was uniform and the change was not apparent in the elastic deformation stage, the plastic deformation stage was mainly analyzed in this paper. In the initial period of plastic deformation, as shown in Figure 11b–d, the strained surface of the specimen was uniformly distributed. As the macro strain gradually increased, the specimen showed a weak inhomogeneity strain distribution, as shown in Figure 11e–i. When the macro strain reached 0.45, the specimen visualized local uneven deformation, and the maximum local strain reached 0.53, as shown in Figure 11j. When the macro strain increased to 0.48, the specimen transformed from uniform plastic deformation to nonuniform plastic deformation, as shown in Figure 11k. The specimen began to enter instability, the overall strain increased, and significant local strain concentration occurred. Finally, the specimen fractured in the red region with the highest local strain. It can be seen from the macro strain distribution that the superalloy sheet had an excellent uniform deformation ability without the assistance of high-frequency pulse current, and the local strain concentration occurred until necking and approaching fracture (Figure 11j). In addition, as shown in Figure 11j,k, the local strain concentration area did not change and further intensified and expanded in the local strain maximum area.

**Figure 12:**
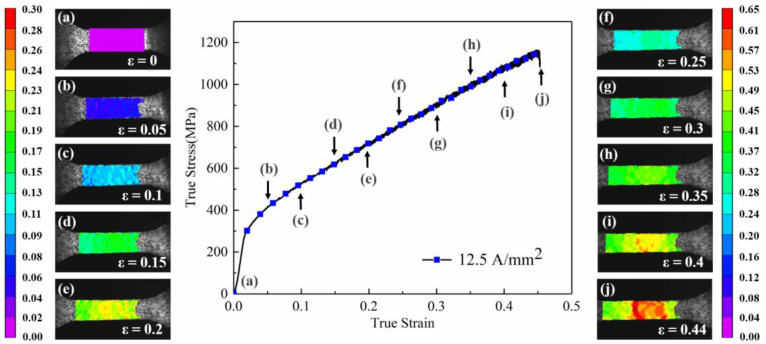
Strain surface distribution along the specimen’s tensile direction with high-frequency pulse current assisted (12.5 A/mm^2^). (**a**–**j**) are the surface distribution figures under macro strain from ε = 0 to ε = 0.44).

The strain distribution along the tensile direction of the specimen assisted by the high-frequency pulse current (current density: 12.5 A/mm^2^) is shown in Figure 12. Similar to the tensile specimen without high-frequency pulse current assisted, the specimen surface distribution was homogeneous, and the change was inconspicuous in the elastic deformation stage. Differently, the high-frequency pulse current-assisted tensile specimen showed a weak strain inhomogeneity in the early stage of plastic strain in Figure 12b–d. As the deformation continued, the local strain concentration occurred earlier near the gauge center, as shown in Figure 12e. When the macro strain ε = 0.2, the maximum local strain was 0.25. When the macro strain was further increased to 0.4, different from the tensile specimen without high-frequency pulse current assisted, the specimen showed a more apparent local strain concentration, as shown in Figure 12i. The maximum local strain was 0.54, which was much higher than the maximum local strain (0.46) of the tensile specimen without the high-frequency pulse current assisted. The specimen entered instability and then the fracture occurred and the macro strain reached 0.44.

Comparing the strain surface distribution of the specimen with the macro strain ε = 0.4 in Figure 11 and Figure 12, there was no obvious strain concentration area in the specimen without the high-frequency pulse current assisted. However, the high-frequency pulse current-assisted specimen showed an obvious local strain concentration and a high strain band in the yellow area. Compared with the specimen without the high-frequency pulse current assisted, the local strain concentration of the specimen assisted by the pulse current appeared prematurely. The local strain concentration was further aggravated with the increase in the macro strain, which caused the earlier instability of the specimen and led to the fracture.

#### 3.2.2. Effect of High-Frequency Pulsed Current on the Strain Line Distribution

**Figure 13:**
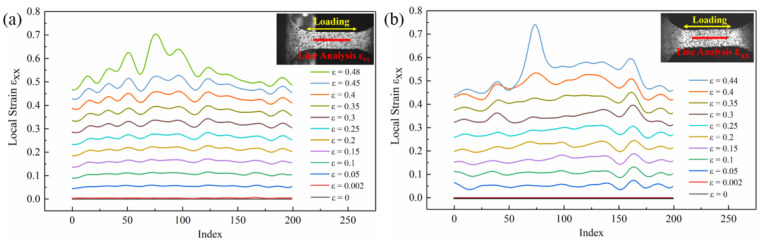
Strain line distribution in the different strain stages along the specimen’s tensile direction: (**a**) without high-frequency pulse current assisted; (**b**) with high-frequency pulse current assisted (12.5 A/mm^2^).

To compare the strain evolution regularity of the two types of specimens more clearly, the strain line distribution of the central area of the tensile specimens gauge was analyzed. A straight line was made along the stretch direction (RD) containing 200 points. As shown in Figure 13, the strain line distribution trend was consistent with the surface distribution. In the elastic deformation stage, the change in the strain of the specimen was not significant, both in Figure 13a,b. As shown in Figure 13a, the strain line distribution was relatively uniform in the early stage of plastic deformation. Before the macro strain ε = 0.25, there was no obvious local strain concentration. As the macro strain increased, the specimen gradually entered the nonuniform plastic deformation stage without the assistance of the high-frequency pulse current (macroscopic strain ε = 0.48). There were three higher peaks in the middle of the strain line distribution. The maximum difference in the local strain between the peak and valley was 0.17. As shown in Figure 13b, compared with the line distribution without the high-frequency pulse current assisted, the specimen assisted by the high-frequency pulse current showed a weaker strain distribution inhomogeneity in the early stage of the plastic deformation. When the specimen entered the nonuniform plastic deformation stage, the strain line distribution appeared had a more obvious peak than the specimen without the high-frequency pulse current. The local strain differential between the peak and the peak valley was 0.29. Therefore, the application of high-frequency pulse current assists more serious local strain concentration, and the unevenness of the strain distribution increased.

The strain line distribution of the specimen without the high-frequency pulse current assisted increased uniformly with the increase in the macroscopic strain. In other words, in addition to entering the local strain concentration area after necking instability, the strain in other linear areas basically uniformly increased. The specimen assisted by the high-frequency pulse current showed an uneven local strain increase trend. Comparing the curves of the macro strains ε = 0.4 and ε = 0.44 in Figure 13b, there was a phenomenon where local curves intersected. This phenomenon indicates that as the macro strain increased, the local strain did not increase but decreased. This represents that the application of the high-frequency pulse current affected the strain evolution of the specimen, resulting in an uneven distribution of the local strain increment of the specimen.

**Figure 14:**
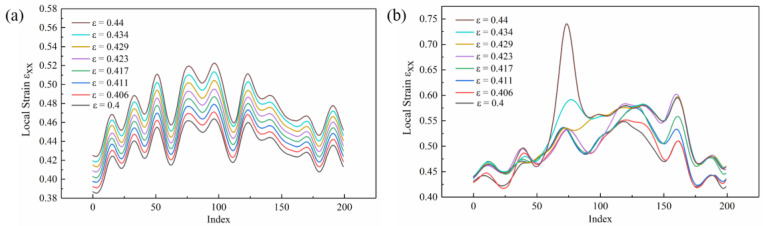
Strain line distribution of the specimen in the strain stage ε = 0.4–0.44: (**a**) without the high-frequency pulse current assisted; (**b**) with the high-frequency pulse current assisted (12.5 A/mm^2^).

The strain line distribution of the specimen without/with the high-frequency pulse current assisted in the macro strain stage ε = 0.4–0.44 are shown in Figure 14a,b, respectively. As the macro strain gradually increased, the strain line distribution of the specimen without high-frequency pulse current assistance increased regularly. The line distributions under the different macro strains did not intersect. The irregularity of the high-frequency pulse current assisted the strain line distribution of the specimen, and the local strain increment of each point in the straight line was not uniform. In addition, the maximum local strain was higher after entering the nonuniform plastic deformation stage. 

**Figure 15:**
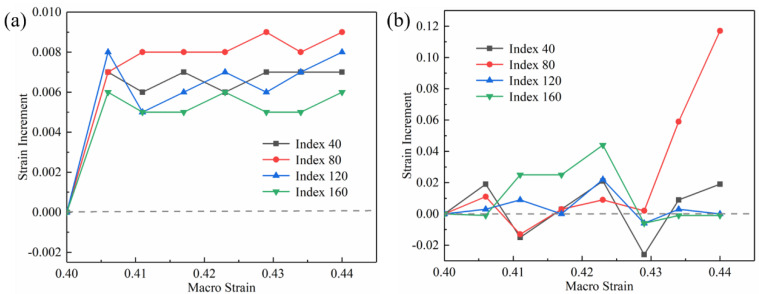
Strain increment: (**a**) without the high−frequency pulse current assisted; (**b**) with the high−frequency pulse current assisted (12.5 A/mm^2^).

The index of 40, 80, 120, and 160 in the lines of Figure 14a,b were selected to represent the increment in the local strain under the increase in the macro strain, as shown in Figure 15. The local strain increment of the tensile specimen without high-frequency pulse current was relatively uniform, as shown in Figure 15a. The local strain increment was both between 0.005 and 0.009 as the macro strain each increased by 0.006. The different local strain increment characteristics appeared in the high-frequency pulse current-assisted tensile specimens. As shown in Figure 15b, the local strain increment changed drastically, and the compressive strain appeared, which is different from the tensile specimen without high-frequency pulse current. The maximum local strain increment was 0.117, which was much larger than the maximum value of 0.009 without the high-frequency pulse current-assisted tensile specimen. The compressive strain appeared in the strain increment of all indexes.

In general, the specimen’s local strain value increased with the macro strain increase during the deformation. However, the compressive strain appeared in the high-frequency pulse current-assisted tensile tests. According to G. H. He et al., the current will produce instantaneous thermal stress (pressure), resulting in reduced dislocation density and enhanced dislocation motion and increasing the linear thermal expansion rate of the specimen [31]. In addition, C. L. Yang et al. reported that electro-pulsing treatment produces inhomogeneous temperature rise and thermal expansion, which can heal voids by selective heating and hot-pressing stress, which shows that the thermal compressive strain will be generated with the assistance of current [32]. Especially in the case of a sizeable macro strain, defects such as voids appear in the specimen. The current targets these defects, resulting in an uneven temperature increase of the specimen and local hot pressing stress. Our team established the fracture mechanism model during the EA tension. The results show that local temperature increase and the local compressive stress will occur when the current passes through the voids [33]. Therefore, with the assistance of the high-frequency pulse current, the local strain value of the specimen will appear to have an inhomogeneous distribution, and the local strain remains unchanged or decreases during the tensile process.

## 4. Conclusions

In this investigation, a high-frequency pulse current-assisted tensile test was carried out on a nickel-based superalloy sheet. The effects of the different high-frequency pulse current density parameters on the mechanical behavior of thin plates were studied in detail. The evolutionary process of the local strain distribution state of the thin plate in high-frequency pulse current-assisted tensile test was studied. The main conclusions are as follows:

1. With the assistance of the high-frequency pulse current, the yield strength and tensile strength decreased significantly. With the increase in the current density, the decrease in the flow stress increased. The phenomenon was mainly caused by the Joule heating effect and the electronic force caused by the applied electric field.
2. The PLC phenomenon appeared in the high-frequency pulse current-assisted tensile test due to the interaction of the diffusion solute atoms on the dynamic pinning and release of moving dislocations. With the increase in the current density, different types of PLC appeared. This was caused by the change in the diffusion (vibrational ability) of the solute atoms under different current density parameters.
3. After applying the high-frequency pulse current, the specimen temperature increased rapidly and increased slightly as the deformation continued. During the tensile test, the Joule heating temperature in the central region of the gauge was always the highest, and there was a local Joule heating effect.
4. With the assistance of the high-frequency pulse current, there was a local uneven strain distribution in the specimen. This was because the current targeted the microscopic defects, resulting in an uneven temperature rise and local hot pressing stress.

## Data Availability

Not applicable.
